# The Effectiveness of Motor Imagery in Balance and Functional Status of Older People with Early-Stage Dementia †

**DOI:** 10.3390/brainsci14111151

**Published:** 2024-11-17

**Authors:** Anna Christakou, Christina Bouzineki, Marousa Pavlou, George Stranjalis, Vasiliki Sakellari

**Affiliations:** 1 Department of Physiotherapy, School of Health Sciences, University of West Attica, 12243 Athens, Greece; 2 Department of Physiotherapy, Laboratory of Biomechanics, School of Health Sciences, University of Peloponnese, 23100 Sparta, Greece; 3 Marousi Day Center Alzheimer Athens Association, 11636 Athens, Greece; cbouzineki@yahoo.com; 4 Centre for Human & Applied Physiological Sciences, King’s College, London WC2R 2LS, UK; marousa.pavlou@gmail.com; 5 Department of Neurosurgery, University of Athens, Evangelismos Hospital, 10676 Athens, Greece; stranjal@otenet.gr; 6 Department of Physiotherapy, University of West Attica, 12243 Athens, Greece; vsakellari@uniwa.gr

**Keywords:** dementia, imagery, physiotherapy, balance, functional status

## Abstract

Background/Objectives: Dementia involves the loss of cognitive abilities and impairs functional abilities in daily life. In motor imagery (MI) techniques, motor acts are mentally rehearsed without any overt body movements. The purpose of the randomized controlled trial was to examine the effects of MI on the motor function of older adults with dementia. Methods: Overall, 160 participants (43 men, 117 women, MMSE M = 23.20, SD = 0.15) from an Athens Day Care Center of the Alzheimer Association were randomized to (a) the MI and exercise group (experimental group) (n = 55), (b) the only exercise group (1st control group) (n = 52) and (c) the neither MI nor exercise group (2nd control group) (n = 53). The exercise session comprised 24 physiotherapy exercise sessions, lasting 45 min each, twice a week for 12 weeks. The exercises were selected from the Otago Exercise Program. Three assessments were performed: (a) one week prior to the program, (b) at one and a half months and (c) after the program. The experimental group performed a 30-minute MI with exercise program content after the end of every physiotherapy exercise session. The Multidirectional Reach Test, Five Times Sit-to-Stand Test (FTSST), Timed Up and Go test (TUG), Functional Gait Assessment (FGA) and Berg Balance Scale (BBS) were used to assess participants’ balance and functional status. Results: In the intention to treat analysis (18 participants dropped out), the 3 × 3 repeated measures ANOVA indicated statistically significant results between the three groups on (a) the TUG (F = 3.06, df (2), *p* = 0.04), (b) the FTSST (F = 3.00, df (2), *p* = 0.05), (c) the forward direction test (F = 4.14 df (2), *p* = 0.02), the lateral right and the lateral left direction tests (F = 3.90, df (2), *p* = 0.02 and F = 7.87, df (2), *p* = 0.00, respectively), and (d) the FGA (F = 4.35, df (2), *p* = 0.01). The Friedman test showed significant statistical significant differences among the three groups for BBS (X^2^ = 7.62, df = 2, *p* = 0.22), and an effect size of partial η^2^ coefficient for F-tests was found. Post hoc comparisons using a Bonferroni test for ANOVA and Wilcoxon test for Friedman indicated that the mean scores for the experimental group and the 1st control were significantly better than the 2rd control group in many dependent variables. Conclusions: The study showed a positive effect of MI on balance and the functional status of older adults with early stages of dementia with possible beneficial effects on maintaining independence and reducing physical decline.

## 1. Introduction

Dementia represents a primarily geriatric syndrome where Alzheimer’s disease is the leading cause and typical form of dementia. The number of people living with dementia worldwide in 2019 was estimated at 57 million and is projected to increase to 153 million by 2050 [1].

It is characterized by a chronic, progressive loss of cognitive function without fluctuating consciousness accompanied by a decline from the individual’s prior level of function and a loss of ability in physical activity, impairing functional abilities in day-to-day life. It confers an increased risk of falls. Each year, 60–80% of people with dementia fall [2]. Indeed, fall rates were higher among people with dementia compared to persons without dementia [3]. Furthermore, studies report that an early stage or mild stage of dementia is associated with an increased falls risk [4], osteoarthritis and pain [5], and poor balance [6], and it represents a stage for early intervention. Another study reports that people with early stage or mild dementia that experienced a progressive memory decline also face self-perceived problems with daily activities, i.e., a decline in memory performance which impairs everyday functioning [7].

Exercise intervention has been used for those with all stages of dementia with inconclusive findings [8]. A recent systematic review revealed that larger studies involving multimodal exercise interventions appear to suggest a positive effect on physical performance [8,9,10] and ADL (activities of daily living) functioning [9,10,11], although not all studies reported a significant difference. These studies incorporated mainly aerobic interventions [9,12,13]. On the contrary, other exercise intervention programs did not improve functioning [14]. Harwood et al. [15] found that the intervention program entitled “Promoting activity, Independence, and Stability in Early Dementia” (PrAISED) did not improve activities of daily living or physical activity, reduce falls, or improve any other secondary health status outcomes in 365 adults with early dementia

The Otago Exercises Program (OEP) is another therapeutic exercise that has been used in the present study; it is a low-cost option that requires minimal equipment. Meta-analyses showed that a 30-minute training session of the OEP significantly improved physical function, pain management, functional independence/functional mobility, static and dynamic balance, and lower limb strength, and it reduced the number/rate of falls in older people [16,17,18]. Similarly, a recently RCT reported that the older people who participated in the OEP for 8 weeks (thrice a week) had better score on Berg Balance Scale than the control group [19]. However, in another RCT, it was found that the OEP did not improve the fear of falling, the strength of the upper extremities and the results of the 6-minute walk test [20]. Due to inconsistent findings, it is crucial to conduct high-quality methodological studies with large and more homogenous samples to determine the effects of exercise programs on health outcomes in participants with dementia.

Harwood et al. [15] suggested using alternative approaches for maintaining function and well-being in people with dementia. Motor imagery (MI) has been used as an alternative and complementary technique which indicates the visual (i.e., imagining ‘seeing’) and kinesthetic (i.e., imagining ‘feeling’) representation of a movement without actual execution [21,22]. Both execution and MI adhere to Fitts’s law, which states that the time to execute or imagine a movement is influenced by the accuracy demands of the task [23,24]. According to the ‘functional equivalence hypothesis’, rooted in motor simulation theory [14,15], the similarities between actual execution and MI arise from shared motor-cognitive neural processes. These processes enable the imagined rehearsal of movement using cognitive motor planning mechanisms [22]. Strong support for the functional equivalence hypothesis comes from brain imaging studies, which show that MI and actual motor execution activate similar brain regions [25,26]. These activations include a distributed premotor–parietal network and several subcortical structures, such as the putamen and cerebellum [25,27].

MI is considered an effective tool in neurological rehabilitation, as evidenced by its use in stroke [28], Parkinson’s disease [29], and multiple sclerosis [30] to improve gait speed, gait performance, balance, and cognitive function, activities of daily living and quality of life. MI has important advantages in that it is not invasive, it is a safe, low-cost therapy, and it can be performed at home [31].

No studies to date have explored the role of MI on the motor function of older adults in the early stages of dementia. Experimental research investigating the psychophysiological mechanisms of MI in dementia is notably lacking. Understanding these processes is not only of theoretical significance but also holds clinical relevance. Such insights could assist physiotherapists in integrating MI into rehabilitation programs, enhancing participants’ mobility. Physical exercise, in combination with MI, may provide preventive benefits by enhancing physical abilities and reducing fall risk, which is crucial for this population. Consequently, this study aims to evaluate the effects of MI on the motor performance of older adults with early-stage dementia.

## 2. Materials and Methods

### 2.1. Study Design

This study was a single blind randomized control trial (RCT) conducted in compliance with the Declaration of Helsinki and Good Clinical Practice (GCP). Ethical Approval was obtained by the Ethics Committee of the University of West Attica (study’s protocol: 93292—26 October 2021). Also, the protocol of the study has been approved by the Day Center Alzheimer Athens Association, Athens, Greece. The trial was registered with ClinicalTrials.gov under the identifier NCT05232526.

### 2.2. Sample

The sample size was determined using G*Power version 3 with an effect size of 0.9, power of 0.8, and an alpha (α) error of 0.05 for an a priori power analysis, which was conducted for a one-tailed *t*-test comparing independent means. A non-probability convenient sampling technique was used. The present study used a total of 160 participants from the Day Care Centers in Athens Alzheimer Association from September 2021 to June 2024, and 142 of them completed the intervention. Older people with early stage of dementia, aged 65 to 95 years, participated. They were allocated to the experimental or to either of the two control groups, using a randomization method by drawing lots. One of the researchers was responsible for the enrollment and assigning participants to the allocation group, the MI and exercise program (experimental group) or to either of the control groups (only exercise program (1st control group), neither MI nor exercise program (2nd control group). The EG followed the 4-week intervention program in addition to their physiotherapy exercise program. The inclusion criteria of the sample were (a) 65 < age < 95 years old, (b) diagnosed with Alzheimer’s type early-stage dementia according to the International Statistical Classification of Diseases and Related Health Problems [ICD]—10 Version: 2019: F00, F01-F03; Mini-Mental State Examination [MMSE] with a score of 20–25 points, (c) good oral and written communication skills and the ability to follow instructions, (d) both sexes, (e) ambulatory, (f) no health issues in the past month and (g) willingness to participate in the study. The exclusion criteria for the sample were (a) late-stage dementia, (b) psychiatric disorders, (c) serious health conditions, such as significant cardiovascular or respiratory disease, (d) co-occurrence of other neurological diseases and (e) not able to walk. An information sheet and consent form was provided to all participants.

### 2.3. Outcomes

Demographic data were collected once in the baseline assessment. The outcomes measures were assessed three times over a period of approximately three months: an initial baseline assessment before the intervention period (pre), a mid-point assessment during the intervention program, and a post-assessment that took place three months after the intervention concluded. The outcomes measures are outlined below.

#### 2.3.1. Balance

(a) The Berg Balance Scale (BBS) is an objective tool used to assess a participant’s ability to safely maintain balance during a series of predetermined tasks. It consists of 14 items, which were each scored on a 5-point ordinal scale ranging from 0 (lowest level of function) to 4 (highest level of function). The assessment takes approximately 20 min to complete. Research has confirmed the reliability of the Berg Balance Scale in older adults with mild to moderate Alzheimer’s disease (AD) [32,33].

(b) The Multidirectional Reach Test (also known as the Reach in Four Directions Test) is a screening tool used to assess participants’ stability limits in four directions: forward, backward, leftward, and rightward. Participants perform a maximal reach in each direction with outstretched arms while keeping their feet flat on the floor, and reach is measured as the total hand excursion using measuring tape [34].

(c) The Five Times Sit-to-Stand Test (FTSST) is a reliable, low-cost tool for assessing sit-to-stand ability. The FTSST records the time taken to stand five times from a seated position as quickly as possible, measuring lower limb strength, balance control, and exercise capacity [35].

#### 2.3.2. Functional Status

(a) The Timed Up and Go Test (TUG) is a simple tool used to investigate a person’s mobility. It measures the time taken for a participant to rise from a chair, walk three meters, turn 180 degrees, walk back to the chair, and sit down, also turning 180 degrees during the process [36,37].

(b) The Functional Gait Assessment (FGA) evaluates a participant’s ability to perform multiple motor tasks while walking. It consists of 10 items, including gait on a level surface, changing gait speed, walking with horizontal and vertical head turns, a 180° pivot turn, stepping over obstacles, walking with a narrow base of support, walking with eyes closed, backward walking, and ascending stairs. Each item is scored on a 4-point ordinal scale (0–3), where 0 indicates severe impairment and 3 indicates normal ambulation. The total score is calculated by summing the scores of all 10 items with a maximum possible score of 30 [38].

### 2.4. Intervention Description

The study included the use of a mental and exercise program including the following.

(a) MI program

MI began as soon as the exercise program started. The experimental group participated in 24 sessions featuring 30 min of imagery practice, which started with the first exercise session. Each imagery session was conducted in a quiet space immediately after the corresponding exercise session. During the imagery sessions, participants visualized the same exercises they had just completed in the exercise program at the Marousi Day Center, Alzheimer Athens Association. All imagery sessions were consistent and identical for all participants in the experimental group.

(b) Physiotherapy exercise program

The participants in the experimental group and the 1st control group completed the exercise program under the guidance of the same experienced physiotherapist at the Marousi Day Care Center in the Athens Alzheimer Association. They received 24 physiotherapy sessions, each lasting 45 min, twice a week, over a period of three months (12 weeks). The exercise program was based on the OEP and included a warm-up period to promote circulation and prepare the body for the exercises. During the warm-up, participants mobilized their joints and stretched their muscles. Afterwards, it included exercises for muscular strength and muscle endurance with and/or without the use of weights. All strength exercises were performed slowly and with control through the subjects’ individual range of movement. Balance is essential for improving posture and performing everyday activities. Dynamic and static balance exercises can enhance confidence and reduce the risk of falling. Cool-down stretches improve flexibility and promote relaxation, helping to reduce fatigue and refresh the body after an exercise session. The exercise program included the following activities: (a) easy marching, (b) head movements, (c) back extensions, (d) ankle movements, (e) front and back knee strengthening, (f) side hip strengthening, (g) calf and toe raises (hold), (h) toe and heel walking, (i) one-leg stances, (j) sideways walking, (k) sit-to-stand, and (l) back-of-thigh and calf stretches All participants perform the same type of exercises during their program. They advanced to the next level of exercises when they were able to complete the current exercises [39].

We examined the imagery ability of the participants of the experimental group by asking them to complete the Vividness of Movement Imagery Questionnaire (VMIQ) [40]. This instrument consists of 24 items related to movement imagery, including visual imagery of movement itself and kinesthetic imagery. Participants are required to imagine performing the movements themselves as well as imagine someone else performing the same movements. The items are grouped into six categories, each with four items: (a) basic movements, (b) basic movements with more precision, (c) movements with control but some unplanned risk, (d) movements involving object control, (e) movements causing imbalance and recovery, and (f) movements requiring control in aerial situations. The VMIQ uses a 5-point scale, ranging from 1 (perfectly clear and as vivid as normal vision) to 5 (no image at all, only a sense of the skill). In the first four sessions of the intervention phase, participants were introduced to imagery and provided with a brief overview of the impact of imagery on clinical and healthy populations. During these initial sessions, each participant engaged in exercises and followed instructions designed to develop their imagery skills in terms of self-perception, vividness, and controllability. This training aims to help participants visualize, control, and vividly construct mental images. Before each imagery training session, a relaxation technique was applied, as it has been shown to enhance the clarity and vividness of imagery representations [41]. At the end of each session, participants completed a manipulation check using a Likert scale ranging from 1 (not at all) to 5 (very much) to assess whether they are imagining the content vividly and accurately.

Assessment was undertaken by the same physiotherapist experienced in the management of the participants with early dementia. The assessor involved in data collection was trained in the study procedures and familiar with the use of the study instruments and measurements. A blind assessor was involved in musculoskeletal assessing of the participants.

### 2.5. Statistical Analysis

Data are expressed as mean ± standard deviation for continuous variables and as frequencies and percentages for categorical variables. The homogeneity between groups was assessed using an independent samples *t*-test for numerical variables and the chi-square (χ²) test for categorical variables with a significance level of α = 0.05. The Kolmogorov–Smirnov test was used for normality analysis of the variables. An intention-to-treat analysis was conducted to account for dropouts, using the last observation carried forward (LOCF) technique [42]. Parametric analyses involved a 3-way repeated measures ANOVA model to compare the time-related changes in the following variables for each group: (a) the TUG, (b) the FTSST, (c) the forward direction test, the lateral right and left direction tests, and (d) the FGA. The effect size for F-tests was assessed using the partial Eta squared coefficient (η²), with values of 0.01, 0.06, and 0.14 representing small, medium, and large effect sizes, respectively. Post hoc comparisons were conducted with Bonferroni’s correction. For non-parametric analyses, Friedman test’s was applied for repeated measures for the BBS test, and post hoc comparisons were performed using the Wilcoxon signed-rank test. All tests were two-sided, and statistical significance was set at *p* < 0.05. All analyses were conducted using the SPSS statistical package (version 29.0.0) (IBM Corporation, Somers, NY, USA).

## 3. Results

Table 1 shows the demographic and clinical characteristics of the participants. One hundred and sixty participants (43 men, 117 women, M = 77.94 years, SD = 7.19, MMSE = 23.20, SD = 0.15) participated in the study. The most common first symptom reported by 137 participants was the loss of memory. No statistically significant differences were found between the three groups at baseline on all the demographic and clinical characteristics of the participants (Table 1).

Eighteen participants dropped out for various reasons [i.e., fractures (2 participants), deaths (7 participants), transportation reasons (5 participants), COVID disease (2 participants), weather issues (2 participants)]; thus, an intention-to-treat analysis due to dropouts was performed using the last observation carried forward (LOCF) technique. The measurements of (a) the TUG, (b) the FTSST, (c) the forward direction test, the lateral right and lateral left direction tests and (d) the FGA were normally distributed, whereas the measurement of BBS were not normally distributed. The 3 × 3 repeated measures ANOVA investigated the differences between the three different groups (between-subjects effect) and across the three repeated measurements (within-subjects effect). Partial variance effect sizes (η^2^) were used to determine the percentage of variation in the data that could be attributed to treatment differences (Table 2). The results showed statistical significant differences between the three groups in all the variables except the back direction test (F = 2, 2.48), *p* = 0.08.

Post hoc comparisons using the Bonferroni correction showed the following mean score trends:

(a) For the TUG test, the experimental group (M = −1.48, SD = 0.60) was significantly different than the 2nd control group (*p* = 0.04);
(b) For the FTSST, the experimental group (M = −1.46, SD = 0.60) was significantly different from the 2nd control group (*p* = 0.05);
(c) For the FUG test, the experimental program (M = 1.09, SD = 0.37) was significantly different from the 1st control group (*p* = 0.01);
(d) For the forward direction test, the 2nd group (M = 6.75, SD = 2.35) was significantly different from the 2nd control group (*p* = 0.01);
(e) For the lateral right direction test, the experimental group (M = −4.73, SD = 1.91) was significantly different from the 1st control group (*p* = 0.04) and the 2nd control group (M = −4.45, SD = 1.71) (*p* = 0.03);
(f) For the lateral left direction test, the experimental group (M = −5.96, SD = 1.30) was significantly different from the 1st control group (*p* = 0.03) and the 2nd control group (M = −8.88, SD = 2.29 (*p* = 0.00).

A comparison of the repeated measures was performed using Friedman’s test showing a significant statistical result between the three groups in BBST χ^2^(2) = 7.63, *p* = 0.02. Post hoc analysis with a Wilcoxon signed-rank test was conducted with a Bonferroni correction applied, resulting in a significance level set at *p* < 0.00. The mean BBST index (±SD) was 53.39 (3.56) at baseline, 54.53 (1.64) in the middle of the intervention program and 54.33 (2.17) at the end of the intervention program. A significant increase was seen between the experimental group and the 1st control group (Z = −4.32, *p* = 0.00) as well as between the intervention group and the 2rd control group (Z = −3.40, *p* = 0.00).

Possible scores on the Vividness of Movement Imagery Questionnaire are from 24 (highest imagery ability) to 120 (lowest ability). The range of scores for ‘watching somebody else’ was 46–90 (M = 60.80, SD = 10.72), and for ‘doing it himself/herself’, the range was 38–78 (M = 50.00, SD = 10.70). The vividness of the majority of images obtained during the ‘doing it himself/herself’ condition was clear and reasonable. The overall imagery ability score ranged from 26.2 to 36.4 (M = 30.76, SD = 2.25). Participants felt the exercise performance fairly to very vividly and clearly.

## 4. Discussion

The aim of the current study was to examine the effect of MI on the motor performance of older people with a neurodegenerative disease, specifically focusing on individuals with dementia. Participants in the experimental group underwent MI sessions following each physiotherapy session. The content of every MI session was identical to that of the OEP session. The study aimed to determine the effectiveness of MI on the balance and functional status of participants with dementia. The results showed that the participants of the experimental group exhibited better balance and functional status than the two control groups as this was recorded by the TUG, FTSST, forward and lateral direction tests, FGA and BBS.

The underlying mechanisms by which MI influences motor behavior remain a topic of debate. MI is a conscious process; that is, it refers to the capability of imagining performing a given motor action or motor skill. It is not so clear yet if this imagining is directly or indirectly correlated to the motor process per se. Evidence suggests that neurophysiological activity during motor imagery closely resembles that seen during actual movement execution. This similarity supports the hypothesis that MI may induce changes in the motor cortex that influence subsequent physical performance. During motor imagery (MI), the motor cortex maintains the same overall population dynamics as during motor execution by reorienting the components related to motor output and/or feedback into a unique, output-null imagery subspace [43]. Specifically, the pathways activated during MI partially overlap with those used in motor execution [44], including the premotor cortex, primary motor cortex, posterior parietal regions (e.g., the inferior and superior parietal lobes), and the cerebellum [45]. The imagery intervention affects the neural information that transferred to the central nervous system. This assumption indicates that imagery plays a role in the cognitive programming of a motor response that takes place in the highest level of CNS function—i.e., motor cortex, basal ganglia, and the cerebellum; thus, imagery is responsible for the adaptive changes in central processes.

Anderson et al. [46] reviewed the use of guided imagery in AD and dementia. They found that only a small number of studies have examined if individuals with dementia can benefit from MI (motor imagery). One such study by Heyn [47] (n = 13) showed improvements in heart rate, overall mood, and engagement in physical exercise following a multisensory intervention that combined imagery with a warm-up session of seated exercise. Additionally, Hussey et al. [48] found that individuals with mild Alzheimer’s could perform basic visual imagery but not at a complex level designed to enhance verbal recognition, which was the aim of the study. As a result, Anderson et al. [46] concluded that due to the limited number of studies on guided imagery interventions for participants with dementia and AD, this modality warrants an evidence grade of C1. Actually, in both of the aforementioned studies of Andersen’s review, the samples had AD and not dementia.

To our knowledge, no studies have directly examined the effects of MI intervention on the functional status of participants with dementia. The psycho-neurophysiological mechanism that takes part in the functional stability of dementia should be further examined. Thus, it is important that new research efforts should be directed to provide additional information on the connection between imagery and the functional stability of participants with dementia.

While research on MI in Parkinson’s disease exists, findings are not similar. A systematic review and meta-analysis, which included 12 studies with 320 participants, assessed the use of MI in rehabilitation outcomes for Parkinson’s disease. In particular, seven studies reported significant improvements in motor function, and one study reported significantly increased confidence in daily task performance. However, no statistically significant effects were found in the meta-analyses due to the small number of studies and the heterogeneity of interventions and outcome measures used [22]. In contrast, a multicenter RCT did not find differences between embedded MI and relaxation with current standard of care in 47 participants with Parkinson disease that followed a six-week intervention period on walking performance, the TUG test, and the 10 m walk test [49]. How et al. [50] discussed in their review the potential use of MI from people with freezing of gait due to Parkinson’s disease and what MI dosage is most effective. Another study showed that imagery training alone enabled the Huntington’s diseases patients to achieve a significant approach to movement isochrony, whereas the Parkinson’s diseases patients showed no marked improvements neither with motor imagery nor with motor practice [51]. MI has also been investigated in patients with multiple sclerosis; Tacchino et al. [52] showed that a few mental repetitions of an action might be sufficient to exert a priming effect on the actual execution of the same action; thus, this can be a promising potential rehabilitation method in this clinical population.

There is considerable evidence supporting the benefits of exercise for individuals with dementia. An international collaborative guideline reported that in people with moderate dementia, exercise could be considered as a means to stabilize disability when compared to usual care [53]. Systematic reviews have showed that both mixed and home-based physical activity improve several non-cognitive outcomes (such as disability and physical function tests) in individuals with dementia [53,54]. An expert consensus statement indicated that 86% of the experts agreed that physical activity/exercise is crucial for maintaining cognitive reserve and function in people with dementia [54]. Li et al. [55] reported that physical exercise improved both cognitive ability and walking ability in patients. They suggested that the number of training sessions should not be excessive with a total of fewer than 30 interventions. The authors recommend long-term exercise training for middle-aged and elderly patients with dementia or those predisposed to it. They specifically recommend multi-component and aerobic training, with two to three exercise sessions per week, each lasting about 60 min. Similarly, in the present study, OPE sessions were performed twice per week for about 45–60 min each.

The present study used a sample with older people with dementia. While many studies have focused on MI’s effects on healthy older adults, few have addressed dementia patients specifically. A systematic review with meta-analyses showed that MI can significantly improve balance gait speed and TUG; however, the quality of evidence was very low to low [56]. Nicholson et al. [57] reported in their RCT that during a single session of motor imagery training, the timing of an imagined locomotor task was refined to better match that of the physically performed task. Motor imagery training led to greater improvements in locomotor performance compared to controls, suggesting that locomotor-related motor imagery can enhance mobility in independent, community-dwelling older adults. A more recent RCT showed that after an exercise intervention three times a week for six weeks, significant improvements were observed in path length, BBS, TUG, velocity, cadence, step length, and stride length in both the motor imagery (MI) training group and the task-oriented training group compared to the control group. These results suggest that motor imagery training combined with functional training has positive effects on balance, gait, and fall efficacy, contributing to fall prevention in the elderly [58]. Similarly, the present study demonstrated that MI can positively affect the motor performance of older adults with early-stage dementia.

A major strength of the current study was its large sample size, which enabled the use of valid tests for the subjective evaluation of balance and functional status. The inclusion of the OEP in this clinical population is also notable. No previous studies have combined OEP with MI for dementia patients. This pioneering research study opens new paths regarding the treatment of dementia. In addition, the imagery ability of the experimental group was accepted. The limitations of the present study are the participants’ diverse age and sex. However, it is not possible to find subjects that have the same demographic characteristics. Also, the participants had a specific stage of dementia, so the present results cannot be generalized to other stages of dementia. Finally, there has been some participant dropout, which is considered normal due to their age.

Future research should be conducted to clarify whether changes in the central nervous system affect muscular performance and if so identify which specific neurophysiological mechanisms are responsible for improvements in balance and functional performance in dementia. Studies should also explore MI’s effects on the middle stage of dementia and if there are differences in the effectiveness between visual and kinesthetic imagery on participants with dementia. It will be interested to investigate in the future the effectiveness of MI on other neurodegenerative diseases and on other variables such as quality of life or the emotional and psychological status of participants with dementia. Also, more valid tests and/or instruments should be used in future studies to assess the MI ability of this clinical population. A replication of the present findings is recommended.

## 5. Conclusions

The current study showed that the participants with early-stage dementia who used MI exhibited improved balance and functional status compared to control groups. These findings suggest that MI may offer new approaches for enhancing dementia treatment. Replicating these results in future studies is essential along with further research into the relationship between MI and conditions such as mild cognitive impairment, dementia, or Alzheimer’s disease. Additionally, future research should explore the underlying psychophysiological processes involved in treating dementia particularly through the use of other complementary techniques.

## Figures and Tables

**Table 1:** Characteristics of the participants.

Variables	Experimental Group (n = 55)	1st Control Group (n = 52)	2nd Control Group (n = 53)	One-Way ANOVA F/X^2^
Age, years M (SD)	79.23 (6.58)	78.46 7.25	76.07 7.50	F (2, 2.87), *p* = 0.06
MMSE	23.45 (2.00)	22.73 2.04	23.41 1.59	F (2, 2.42), *p* = 0.09
*Education*				χ^2^ (2, N = 160) = 1.27, *p* = 0.28
University, n (%)	32 (58.2)	23 (44.2)	24 (45.3)	
High School, n (%)	14 (25.5)	16 (30.8)	18 (34)	
*Family status*				χ^2^ (2, N = 160) = 0.19, *p* = 0.82
Married n (%)	35 (63.5)	33 (63.5)	33 (62.3)	
Widow n (%)	17 (30.9)	13 (25)	17 (32.1)	
Divorced	1 (1.8)	1 (1.8)	2 (3.8)	
*Live together*				χ^2^ (2, N = 160) = 0.14, *p* = 0.86
Husband/Wife	34 (61.8)	35 (67.3)	33 (62.3)	
Child	11 (20)	8 (15.4)	6 (11.3)	
Take care	-	3 (5,8)	1	
Alone	10 (18.2)	6 (11.5)	10 (18.9)	
*Have children*				χ^2^ (2, N = 160) = 2,87, *p* = 0.06
Yes	53 (96.4)	52 (100)	44 (83)	
No	2 (3.6)	-	9 (17)	
*Number of children*				F (2, 2.67), *p* = 0.07
1 child	14 (25.5)	13 (25)	23 (43.4)	
2 children	33 (60)	32 (61.5)	19 (35.8)	
*Profession*				χ^2^ (2, N = 160) = 2,37, *p* = 0.09
Civil servant	10 (18.2)	5 (9.6)	6 (11.3)	
Teacher	7 (12.7)	7 (13.5)	9 (17)	
Housework	6 (10.9)	5 (9.6)	7 (13.2)	
Private servant	5 (9.1)	16 (30.8)	6 (11.3)	
Chief engineering in military navy	3 (5.5)	1 (1.9)	1 (1.9)	
Salesman	1 (1.8)	-	-	
*Falls*				F (2, 0.99), *p* = 0.37
No falls	32 (58.2)	33 (653.5)	33 (62.3)	
1 fall	14 (25.5)	10 (19.2)	16 (30.2)	
2 falls	8 (14.5)	9 (17.3)	4 (7.5)	

The italics are the demographic characteristics of the participants.

**Table 2:** Descriptive statistics and 3 × 3 ANOVA test of the TUG, FTSST, multiforward, lateral right and left direction tests and FGA (mean, SD).

Variables Measurement (M, SD)	Experimental Group (n = 55)	1st Control Group (n = 52)	2nd Control Group (n = 53)	F (2, df)	*p*-Value	η^2^
TUG						
1st	11.07 (3.11)	10.98 (3.00)	10.96 (4.03)	16.85	0.00	0.18
2nd	10.38 (2.48)	10.78 (2.95)	11.52 (4.23)			
3rd	9.85 (2.94)	11.04 (3.32)	13.24 (4.08)			
FTSST				19.26	0.00	0.19
1st	13.84 (3.97)	13.60 (4.61)	13.21 (3.85)			
2nd	12.70 (2.90)	12.65 (3.55)	13.85 (3.65)			
3rd	11.32 (2.23)	12.63 (3.33)	15.18 (3.84)			
Forward Direction				4.14	0.02	0.05
1st	57.50 (16.93)	59.79 (63.76)	53.79 (13.38)			
2nd	62.19 (15.47)	63.76 (17.89)	55.58 (10.56)			
3rd	72.31 (14.78)	80.31 (16.86)	74.23 (18.42)			
Lateral Right Direction				3.90	0.02	0.05
1st	49.76 (10.43)	54.75 (14.26)	55.69 (11.73)			
2nd	52.49 (10.95)	55.35 (16.24)	58.68 (11.36)			
3rd	53.81 (10.07)	60.16 (13.34)	55.05 (11.32)			
Lateral Left Direction				7.86	0.00	0.09
1st	48.13 (11.66)	54.37 (13.29)	61.33 (12.75)			
2nd	53.65 (11.61)	57.30 (14.26)	61.77 (12.09)			
3rd	72.30 (14.78)	80.30 (16.86)	77.62 (15.29)			
FGA				4.43	0.00	0.05
1st	28.81 (2.22)	28.34 (2.48)	29.17 (1.28)			
2nd	28.78 (2.06)	27.38 (3.34)	28.07 (2.23)			
3rd	29.09 (1.65)	27.69 (4.07)	27.26 (2.40)			

## Data Availability

Dataset available on request from the authors.
